# A Genome-Wide Investigation of Expression Characteristics of Natural Antisense Transcripts in Liver and Muscle Samples of Pigs

**DOI:** 10.1371/journal.pone.0052433

**Published:** 2012-12-20

**Authors:** Congying Chen, Rongxing Wei, Ruimin Qiao, Jun Ren, Hui Yang, Chenlong Liu, Lusheng Huang

**Affiliations:** 1 Key Laboratory for Animal Biotechnology of Jiangxi Province and the Ministry of Agriculture of China, Jiangxi Agricultural University, Nanchang, People’s Republic of China; 2 Nanchang Working Canine Base, Nanchang, People’s Republic of China; Kyushu Institute of Technology, Japan

## Abstract

Natural antisense transcripts are endogenous transcripts that are complementary to the sense-strand of DNA. These transcripts have been identified in various eukaryotic species and are involved in a broad range of regulatory events and biological processes. However, their general biological functions, expression characteristics and regulatory mechanisms are still unclear. In this study, 497 liver and 586 muscle samples were harvested from a White Duroc×Erhualian F_2_ resource population. The expression profiles of sense and antisense transcripts were determined by tag-based RNA sequencing. We identified 33.7% and 20.4% of transcripts having both sense and antisense expression, and 12.5% and 6.1% of transcripts only expressing antisense transcripts in liver and muscle, respectively. More than 32.2% of imprinting or predicted imprinting genes in the geneimprint database were detected with both sense and antisense expression. The correlations between sense and antisense expression in sense-antisense pairs were diverse in both liver and muscle, showing positive, negative or absent correlation. Antisense expression increases gene expression variability. More interestingly, compared to eQTL mapping of sense transcripts in which more than one eQTL was mapped for a transcript, only one eQTL was identified for each antisense transcript, and the percentage of *cis*-eQTL in antisense eQTL was higher than that in sense eQTL. This suggests that the expressions of antisense transcripts tend to be *cis-*regulated by a single genomic locus. To our knowledge, this study is the first systematical investigation of antisense transcription in pigs. The findings improve our understanding of the complexity of porcine transcriptome.

## Introduction

The breakthroughs in the field of mammalian genome and transcriptome in recent years have shed light on transcriptome complexity. Multiple new classes of RNAs have been uncovered, including antisense transcripts. Natural antisense transcripts (NATs) are endogenous transcripts that are complementary to the sense-strand of DNA either at a same locus (*cis*-NATs) or a different locus (*trans*-NATs) [1], [2]. *Cis* -NATs display perfect sequence complementarity to their genomic overlap and target their sense RNAs in a one-to-one fashion, whereas a single *trans-*NAT may target several sense transcripts [3]. According to the classification schemas by Zhang et al. (2006), *cis-*NATs can be categorized into six categories of divergent (head-to-head), convergent (tail-to-tail), full overlap, contained, intronic and others. The tail-to-tail orientation is the most prevalent [4].

The generation mechanism of NATs is largely unknown. A recent study reported that antisense transcripts typically originate from bidirectional promoters shared with divergent genes [5]. NATs were first detected in viruses [6]. Recently, with the technological development of microarray and sequencing, an increasing number of NATs has been identified in eukaryotic organisms, including human [7], [8], mice [9], [10], rats [10], chicken [10], drosophila [11] and rice [12]. More than 20% of human genes have antisense transcripts [7]. The percentage of antisense transcripts in transcriptome of mice and drosophila was 29% and 15%, respectively [9], [11]. In plants, the first large-scale study of NATs was reported in rice [12], where 7% of transcripts were detected as antisense transcripts. More recently, 25.7% of tags in wheat were discovered to overlap with antisense transcripts by serial analysis of gene expression tags (SAGE) [13]. But to our knowledge, no report was found for systematic and genome-wide study of NATs in pigs.

Although the general biological functions and regulatory mechanisms of NATs are still unclear, it has been proven that NATs can participate in a broad range of regulatory events, such as RNA interference (RNAi) [14], alternative splicing [15], mRNA processing [16], RNA stability and translation [17], genomic imprinting [18], DNA methylation [19] and X-chromosome inactivation [20]. More and more validated antisense transcripts have been confirmed to relate to various human disorders. For examples, a non-coding RNA antisense to moesin at human chromosome 5p14.1 is associated with autism [21]; *cis*-antisense of *GDNF* gene relates to Alzheimer disease [22]; over-expression of the natural antisense *hypoxia-inducible factor-1 alpha* transcript is associated with malignant pheochromocytoma [23]. However, the number of experimentally validated NATs is still limited and a majority of unannotated NATs remain to be uncovered for their biological roles.

In this study, we used the tag-based RNA sequencing (digital gene expression, DGE) to analyze expression profiles of genome-wide sense and antisense transcripts in two porcine tissues of liver and muscle, a metabolically active tissue that is critical to body composition, obesity and carcass traits in pigs, and a main organ for meat production, respectively. All samples were harvested from F_2_ individuals in a large scale White Duroc×Erhualian intercross. We analyzed the distribution of antisense transcripts in porcine transcriptome, discussed the expressions of antisense transcripts in imprinting genes. Especially, the large sample size allowed us to accurately evaluate the correlation between sense and antisense expression in sense-antisense pairs. Furthermore, we performed the expression quantitative trait loci (eQTL) mapping for antisense transcripts using the genotyping data from Porcine SNP60 BeadChip. To our knowledge, this study is the first systematical investigation of antisense transcription in pigs. The findings improve our understanding of the complexity of porcine transcriptome.

## Results

### Identification of Widespread Antisense Transcripts that were Validated by Strand-specific qRT-PCR

**Figure 1:**
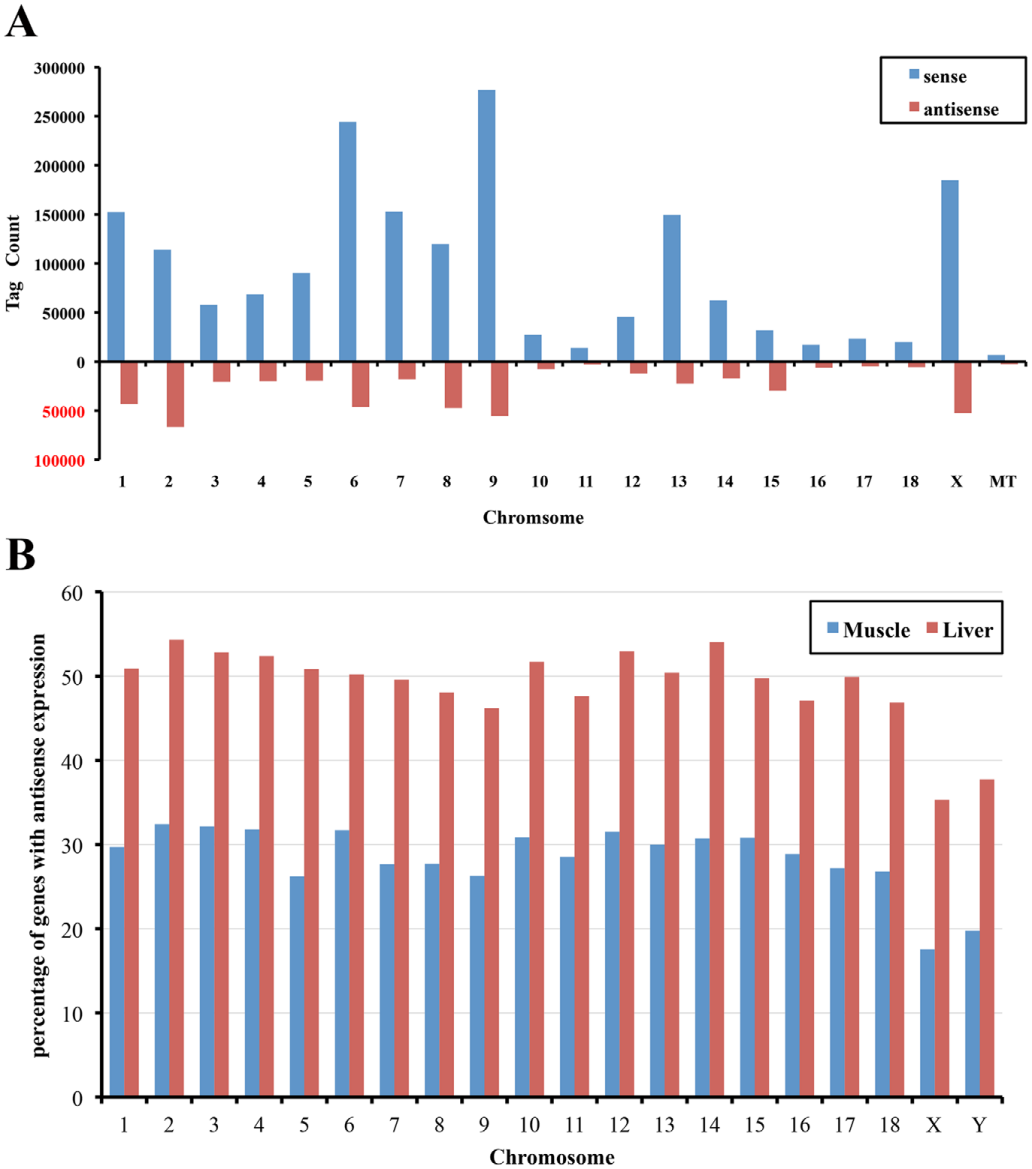
The chromosomal distribution of antisense transcripts. (A) The histogram indicates the tag distribution of sense and antisense transcripts on each chromosome shown in the *X*-axis. The *Y*-axis shows tag counts of sense and antisense transcripts. (B) The figure indicates the percentage of genes with antisense transcription (*Y*-axis) on each chromosome (*X*-axis).

To assess genome-wide antisense transcription, we constructed the cDNA libraries for high-throughout next generation tag sequencing by Illumina GAII with 497 liver and 586 *longissimus dorsi* muscle samples from a F_2_ intercross population. Gene expression profiles were generated by sequencing ‘CATG’ tags. The average raw tag numbers were 6 and 5 million reads (ranged from 3 to 10 million reads) for liver and muscle, respectively. Clean tags accounting for 93.6%–98.8% (average 96.4%) of raw tags were used for further analysis. The clean tags were mapped to the swine genome assembly 10.2 and the pig transcript units from Unigene and PEDE dataset. We were able to map 84.2% (for liver) or 81.3% (for muscle) of clean tags to pig transcripts. Overall, about 14.0% of clean tags were mapped to the minus strand, which were significantly less than sense tags (Figure 1A). These mapped clean tags were used for further detecting antisense transcripts in porcine transcriptome.

Those transcripts with both sense and antisense tag numbers <5 were discarded from further analysis. Clean tags corresponded to 37,229 and 42,327 distinct swine transcripts in liver and muscle, respectively. We assessed the expression level of each transcript by counting the total number of clean tags matching a transcript, and the expression level was normalized to tag number of each transcript per million clean tags (TPM). Referring to the classification schemas by He et al. (2008) [24], for antisense transcription, transcripts could be divided into three main classes. Sense transcripts (or sense genes) were defined as those with distinct antisense tags <5 reads and distinct sense tags >5 reads; antisense transcripts (or antisense genes) were defined as those with distinct sense tags <5 reads and distinct antisense tags >5 reads. The sense-antisense transcripts (or sense-antisense pairs/genes) included the remaining genes, all of which contained both sense and antisense tags with >5 reads. In liver, we detected 12,586 (33.7%) sense-antisense transcripts, 20,033 (53.7%) sense transcripts and 4,680 (12.5%) antisense transcripts among 37,299 detected transcripts. The number of sense-antisense transcript, sense transcript and antisense transcript among 42,327 detected transcripts in muscle was 8,641 (20.4%), 31,104 (73.5%) and 2,582 (6.1%), respectively (Table 1). Significantly higher percentage of antisense transcripts was detected in liver than that of in muscle. Moreover, 9,547 transcripts had antisense transcription both in liver and muscle.

**Table 1:** Classification of genes with respect to antisense transcription.

	liver		muscle	
	No. ofgenes	Fraction	No. ofgenes	Fraction
sense-antisense genes	12,586	33.7%	8,641	20.4%
sense genes	20,033	53.7%	31,104	73.5%
antisense genes	4,680	12.5%	2,582	6.1%
total	37,299		42,327	

We found that the percentage of transcripts having antisense transcription on each autosome is similar, ranging from 26.2% to 32.4% in muscle and 46.2% to 54.3% in liver. Over 50% of genes on chromosomes 1, 2, 3, 4, 5, 10, 13 and 14 encode antisense transcripts in liver, showing the widespread of antisense expression in pigs (Figure 1B). The chromosome X contains the fewest genes with antisense transcription (17.6% in muscle and 35.3% in liver).

We randomly selected 7 genes including *alcohol dehydrogenase 4 (ADH4), complement C1q subcomponent subunit B (C1QB), carbonic anhydrase 3 (CA3), eukaryotic initiation factor 4A-II (EIF4A2), glycine amidinotransferase (GATM), insulin-like growth factor 2 receptor (IGF2R)* and *LIM domain-binding protein 3 (LDB3)* to validate their sense and antisense expression by strand-specific qRT-PCR. The correlation coefficients between values of sense/antisense ratio obtained by qRT-PCR and DGE were calculated. The coefficients were 0.99, 0.71, 0.96, 0.92, 0.99, 0.98 and 0.98 for *ADH4, C1QB, CA3, EIF4A2, GATM, IGF2R* and *LDB3*, respectively. This result confirmed the high reliability of expression levels of sense and antisense transcripts detected by DGE.

### Functional Annotation of Genes with Antisense Transcription

A number of studies have indicated that eukaryotic antisense RNAs are involved in the regulation of gene expression of the corresponding sense RNAs at various levels, such as transcription, mRNA processing, splicing, stability, transport and translation [17], [25], [26]. To address the hypothesis that genes with antisense transcription might include transcriptional regulators and signaling molecules, we investigated which term of biological process, molecular function or cellular component is likely to be more annotated to these genes by gene ontology (GO) annotation. We identified antisense and sense-antisense transcripts enriched (corrected *P*-value <0.05) in many GO terms than expected (Table S1). In the cellular component category, top three terms annotated to the antisense and sense-antisense transcripts were intracellular (*P* = 6.4×10^−256^), cytoplasm (*P* = 4×10^−226^) and membrane-bounded organelle (*P* = 2.7×10^−185^). Among GO molecular functions, the two most overrepresented ones were protein binding (*P* = 1.9×10^−114^) and catalytic activity (*P* = 4.4×10^−81^) (Table S1). In addition, the terms of nucleotide binding, RNA binding, translation factor activity, nucleic acid binding, transcription factor binding, RNA polymerase II transcription factor activity and translation initiation factor activity were significantly enriched (corrected *P*-value <0.05). These function terms are known to be relevant to the process of gene expression regulation.

### Many Imprinting Genes have Antisense Transcription

Several studies have reported that sense-antisense pairs are universally associated with imprinted loci [9], [27]. In this study, we downloaded all 239 imprinting or predicted imprinting genes of 7 mammals in the geneimprint database and examined the relative abundance of the antisense expression of these imprinting genes in porcine liver and (or) muscle. We found that 77 (32.2%) imprinting genes had both sense and antisense expression, of which 47 showed sense and antisense expression in both liver and muscle, 3 only in muscle and 27 only in liver. The normalized expression levels of sense and antisense transcripts of these imprinting genes in liver and muscle are listed in Table S2. Overall, large standard deviations were observed on expression levels of both sense and antisense transcripts of these imprinting genes. Five out of 11 porcine imprinting genes listed in the geneimprint database exhibit the antisense transcription in this study including *IGF2*, a well-known paternal imprinting gene in pigs [28].

### Correlation between Sense and Antisense Expression of Transcript

With the large sample size in this study, we evaluated the correlation between sense and antisense expression of each sense-antisense pair. In total, 2,441 sense-antisense pairs expressed in all 497 liver samples and 2,544 sense-antisense pairs in all 586 muscle samples were used to calculate the correlation coefficients between their sense and antisense expression. In livers, high correlations were observed in 15.6% of sense-antisense pairs (| *r* | ≥0.5), 24.3% had the moderate correlations (0.5> | *r* |≥0.3), and 60.1% showed low or no correlations (| *r* |<0.3). In muscles, the percentage of sense-antisense pairs showing high, moderate and low correlation was 14.3%, 24.7% and 61.0%, respectively (Table 2). There were 29.4% and 41.9% of sense-antisense pairs having negative correlation between sense expression and its antisense counterpart in liver and muscle, respectively.

**Table 2:** The distribution of correlation coefficients between sense and antisense expression in liver and muscle.

	1> r≥0.5	0.5>r≥0.3	0.3>r≥0	0>r≥−0.3	−0.3>r≥−0.5	−0.5>r≥−1	total
liver	312 (12. 8%)	436 (17.9%)	974 (39.9%)	494 (20.2%)	157 (6.4%)	68 (2.8%)	2,441
muscle	261 (10.3%)	407 (16.0%)	808 (31.8%)	745 (29.2%)	221 (8.7%)	102 (4.0%)	2,544
Both in live and muscle	68	77	233	133	23	11	545

**Figure 2:**
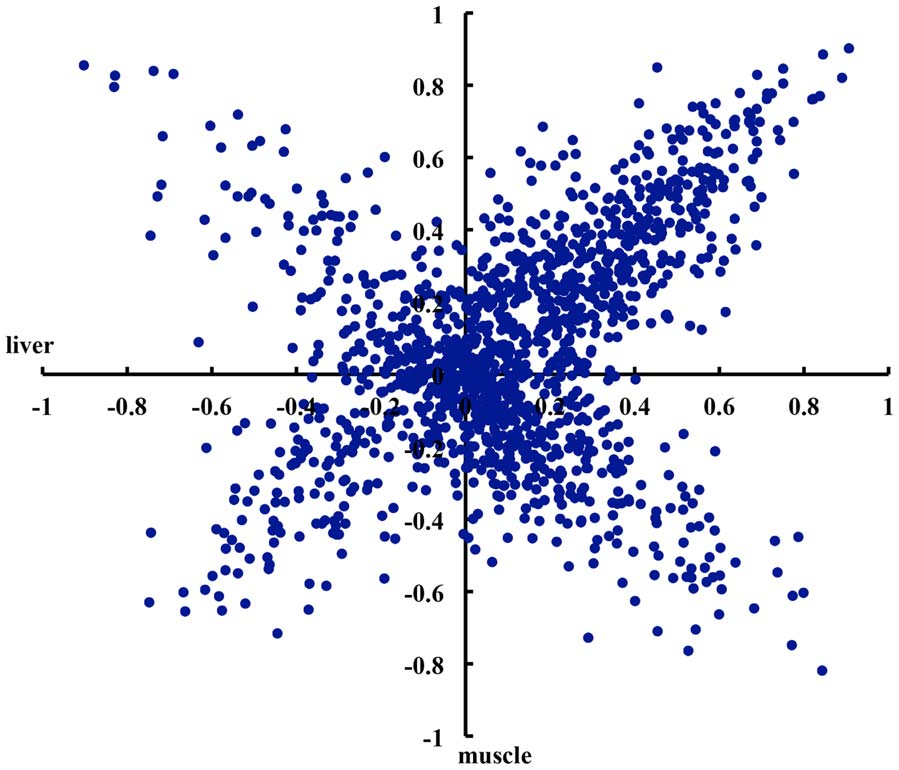
The correlation between sense and antisense expression of transcripts. The distribution of correlation coefficients between sense and antisense expression in 1,396 sense-antisense pairs in both liver and muscle. The *X* and *Y*-axis indicate the distribution of correlation coefficients of 1,396 sense-antisense pairs obtained in liver and muscle, respectively.

To investigate whether the correlation between sense and antisense expression was a tissue-specific event, we analyzed 1,396 sense-antisense pairs that had sense and antisense expression in both liver and muscle. Of the 1,396 sense-antisense pairs, 545 showed the similar correlations in both tissue samples. Moderate to high positive correlations were observed in 145 out of 545 sense-antisense pairs (*r*≥0.3) and high negative correlations were detected in 34 pairs (*r*≤−0.3) (Table 2). The correlations in other 851 sense-antisense pairs showed the apparent tissue-specificity. There were 62 sense-antisense pairs whose sense and antisense expression showed moderate to high positive correlation in liver while negative correlation in muscle. Conversely, 42 pairs exhibited negative correlation in liver but positive correlation in muscle. In addition, significant correlations between sense and antisense expression were detected for 273 sense-antisense pairs in only one of two tissues (Figure 2). These diverse correlations indicate the complex regulation mechanism of sense and antisense expression. As a proof of principle, the correlation coefficient between the expression levels of *IGF2* and its antisense counterpart *IGF2-AS* was −0.39 in muscle (*P* = 2.2×10^−16^). This suggested that the antisense expression of *IGF2* had discordant de-repression of sense transcript expression. It is consistent with the previous report that the sense transcript of *IGF2* can interfere with *IGF2-AS* transcript in muscle [29]. But this significant correlation was not found in liver (*r* = 0.02), indicating the existence of tissue-specific interference.

### Antisense Expression Increases Gene Expression Variability

**Figure 3:**
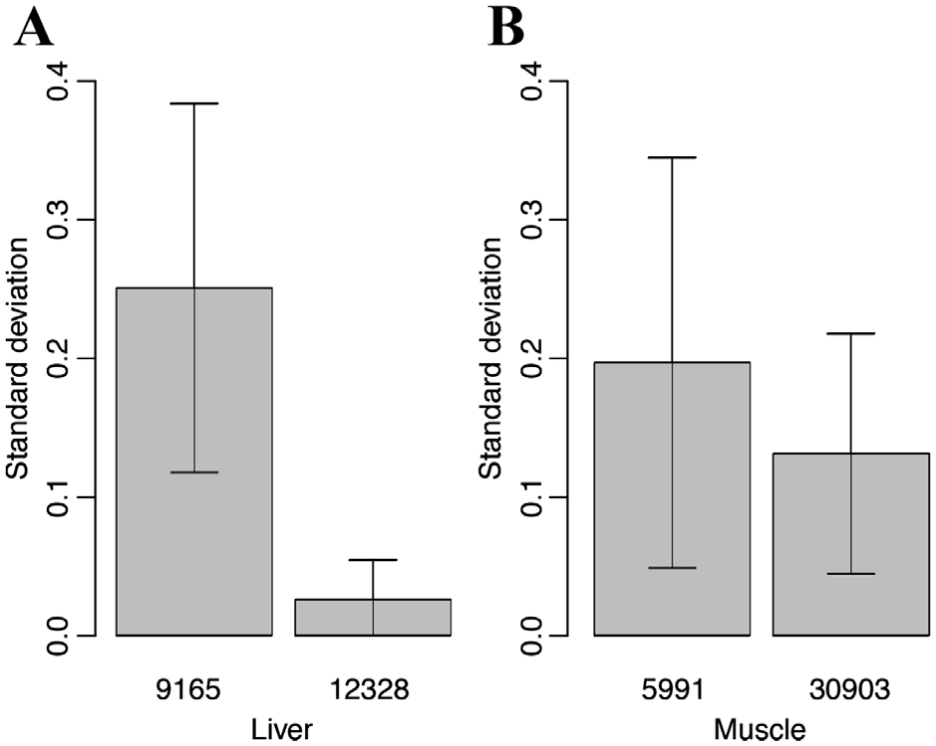
Antisense expression associates with larger gene expression variability. The histograms (A) and (B) show the gene expression variability of transcripts with and without antisense expression in liver and muscle, respectively. The *X*-axis indicates the sample number and the *Y*-axis shows the standard deviation of gene expression levels.

To evaluate the interference of antisense transcript on gene expression in pigs, we compared the expression variability of genes with antisense expression to that of genes without antisense expression. Total 9,165 and 5,991 sense-antisense pairs in livers and muscles were analyzed for the gene expression variability with 12,328 and 30,903 sense transcripts as controls. We found that the expression variation of genes with antisense transcripts was apparently larger than that of genes without antisense expression in both liver and muscle. The standard deviations of gene expression levels of sense-antisense pairs and sense transcripts were 0.25±0.13 *vs.* 0.03±0.03 and 0.20±0.15 *vs.* 0.13±0.09 in liver and muscle, respectively (Figure 3). This result indicates that antisense expression associates with a larger dynamic range of gene expression.

### eQTL Mapping of Antisense Transcript

To map the genomic region influencing antisense transcript expression (antisense transcript eQTL mapping), all White Duroc×Erhualian F_2_ animals with sense and antisense transcript expression data were genotyped with Porcine SNP60 BeadChips (Illumina). More than 37,000 SNPs passed the quality check (QC) based on their call rates, minor allele frequencies and HWE test statistics. The 2,441 and 2,560 sense-antisense transcripts detected in all 497 liver or 586 muscle samples were selected for further antisense eQTL mapping. At false discover rate (FDR) <5% (corresponding *P*–value = 4.85×10^−6^), a total of 370 and 399 antisense eQTL were identified in liver and muscle, respectively (Table S3). Interestingly, unlike eQTL mapping of sense transcripts, only one eQTL was mapped for one antisense transcript. We defined eQTL locating within less than 5 Mb from the target antisense transcript as *cis*-antisense eQTL, otherwise, those eQTL were classified as *trans*-antisense eQTL. In liver, we detected 219 *cis*-antisense eQTL and 110 *trans*-antisense eQTL. The numbers of *cis*- and *trans*- antisense eQTL in muscle were 226 and 130, respectively (Table 3). Because the chromosomal locations of some transcripts or SNPs can not be unambiguously determined with the current reference genome assembly (build 10.2), the acting ways of 41 and 43 antisense eQTL in liver and muscle were unknown.

**Table 3:** Numerical summary of the whole-genome antisense eQTL mapping.

tissue	animal	transcript	SNP	antisense eQTL	*cis*-antisense eQTL	*trans*-antisense eQTL	Overlapped sense eQTL (<2 Mb)
liver	497	2,441	37,540	370	219	110	76
muscle	586	2,560	37,288	399	226	130	44

A genome-wide sense eQTL mapping was performed in our previous study (unpublished data). To examine whether some chromosomal regions regulate both sense and antisense expression of sense-antisense transcripts, we comprehensively analyzed the eQTL mapping results of both sense and antisense transcripts. There were only 133 and 107 sense-antisense transcripts in liver and muscle, respectively, having both sense and antisense eQTL. The antisense eQTL for 76 and 44 out of these 133 and 107 sense-antisense transcripts were overlapped with their sense eQTL (the distance between sense and antisense eQTL was <2 Mb, Table S4). This result suggests that the expression regulation of antisense transcript is different from the sense transcript.

## Discussion

A widespread prevalence of antisense transcription has been reported in many species. In this study, we used high-throughout next generation tag sequencing to determine the genome-wide antisense transcription in pigs. Because this 3' tag digital gene expression analysis used oligo-dT priming for first strand cDNA synthesis and generated libraries that were enriched in the 3' untranslated regions of polyadenylated mRNAs [30], [31], the category of antisense transcription analyzed in this study was *cis* natural antisense transcripts. The estimated fractions of genes associated with the antisense transcription in mammalian cells vary from 2% to more than 70% of the total genes [9], [24]. We found that more than 26.5% of transcripts had the antisense transcription in porcine muscle. This percentage is similar to that of in mouse (28.7%) and wheat genome (25.7%) [9], [13], but higher than that of in human cells (10.4%–18.4%) [24]. A significantly higher percentage (46.2%) of transcripts was found with antisense transcription in porcine liver. Unlike the situation in the mouse genome where antisense transcripts are unevenly distributed [9], porcine autosomes have the similar percentages of transcripts with antisense transcription. But just like in human and mice [4], [9], the antisense transcripts are significantly less prevalent on chromosome X. The X-inactivation in mammals is a possible cause [32].

The GO terms of RNA processing, RNA splicing, mRNA metabolic process and gene expression in biological process were significantly overrepresentation. This result supported the previous findings that eukaryotic antisense RNAs regulate the transcription through mRNA processing, splicing, stability, transport and translation [17], [25]. The most overrepresented GO terms of cellular component, molecular function and biological process were consistent with the previous findings [4], [9]. Antisense transcripts are involved in diverse biological functions through its coding proteins or regulating the gene expression [33]. The growing list of validated sense-antisense transcripts includes many important developmental genes or various human disorder-associated genes. Some sense-antisense pairs identified in this study have been reported to associate with human disease to date. For examples, both sense and antisense expression were identified for *apolipoprotein E* gene (*ApoE*) in porcine liver in this study. The antisense transcript of *ApoE* is conserved between human and mouse. It perhaps involves in *ApoE* expression and has been linked to the familial onset form of Alzheimer’s disease [34]. *Fragile X mental retardation 1* gene (*FMR1*) also showed the antisense transcription in porcine liver. In humans, the antisense transcript of *FMR1* has an antiapoptotic function and might contribute to fragile X syndrome [35]. Other genes such as *IGF2R*, *guanine nucleotide binding protein, alpha stimulating* (*GNAS*), *myelin basic protein* (*MBP*) and *Topoisomerase 1* (*TOP1*), that have antisense expressions in pig liver or muscle are associated with tumor suppressor [36], signal transduction [37], myelin formation [38] and cell cycle [39] in human or mouse.

*Cis*-natural antisense transcripts have been implicated in imprinting as an important regulatory mechanism [32], [36]. Several reports suggested that sense-antisense pairs are universally associated with genomic imprinting [9], [40], [41]. The percentage of imprinted genes that are associated with antisense transcription ranged from 15.0% [42] to 81.0% [9] in mammals. In this study, the antisense transcription was detected in total 32.2% (77) of imprinted genes deposited in the geneimprint database. This finding was consistent with that reported by Zhang et al. (2006), in which 24.0 to 47.0% of human and mice imprinted genes were sense-antisense pairs [4]. Of these 77 imprinting genes, 30 are paternal imprinting gene, 44 are maternal imprinting gene and 3 are isoform dependent. No significant bias of natural antisense expression was observed on paternal or maternal imprinting genes.

Natural antisense transcripts could play positive or negative regulation roles at different stage of gene expression. Chen et al. (2005) found that sense-antisense pairs tend to be co-expressed or inversely expressed more frequently than would be expected by chance [43]. The basal expression levels of sense and antisense transcripts in different tissues and cell lines can be either positively or negatively correlated in mice or humans [9], [24]. Gyorffy et al. (2007) observed an inverse correlation between sense and antisense expression at high expression value and found that the higher the gene expression, the stronger correlation was observed [44]. In this study, the correlation between sense and antisense expression was diversified in different sense-antisense pairs and tissues. This result was consistent with the previous findings in human and mouse where antisense transcript expression in different human cell lines is not always linked to the expression of sense gene [9], [24], [45]. The diverse correlations between sense and antisense expressions suggested that the regulation mechanisms of antisense transcription might be complex. Four mechanisms of transcriptional interference, RNA masking, double-stranded RNA dependent mechanism and chromatin remodeling have been well documented at present [1]. We also found that the transcripts with antisense transcription had the increased gene expression variability. This result was similar to that obtained in *Saccharomyces cerecisiae* [46]. The large gene expression variability may be result of co-regulation of sense and antisense transcript of the sense-antisense pair. These findings indicate that antisense-mediated regulation of gene expression must operate through a variety of mechanisms, and that antisense transcripts are a heterogeneous group of regulatory RNAs [33].

Braunschweig et al. (2004) reported that *IGF2* antisense transcript expression in porcine postnatal muscle is affected by a quantitative trait nucleotide in intron 3 [29]. In this study, a large scale F_2_ resource population with antisense transcript and 60K SNP data allowed us to map the genomic region influencing the expression level of antisense transcript. The percentage of antisense transcripts that were mapped the eQTL was similar to that of sense transcripts (15.2% *vs.* 16.6% in liver). Interestingly, compared to the eQTL mapping of sense transcripts, there were several distinct characteristics in the antisense eQTL mapping: 1) only one antisense eQTL was mapped for an antisense transcript. However, an average of 1.87 eQTL were identified for a sense transcript with a range of 1 to 19 eQTL; 2) the percentage of *cis*-antisense eQTL was 59.2% and 56.6% in liver and muscle, respectively (Table S3). However, there were only 28.0% and 23.5% of *cis-*eQTL in sense eQTL mapping in liver and muscle; 3) there were very few antisense eQTL (76 and 44) overlapping with the sense eQTL. This suggested that the expression of sense and antisense transcript was regulated by different molecular mechanisms. The antisense transcription tends to be *cis*-regulated by a single genomic region.

### Conclusions

A proportion (26.5% and 46.2%) of transcripts has the antisense transcription in porcine muscle and liver. About 32.2% of imprinting genes show both the sense and antisense transcript. The correlation between sense and antisense expression of sense-antisense transcripts is diverse with positive, negative or absent correlation. Antisense expression increases gene expression variability. eQTL mapping of antisense transcripts indicates that the expression of sense and antisense transcript is regulated by different molecular mechanisms. The findings would significantly improve our understanding of the complexity of porcine transcriptome, and provide a comprehensive view of genome-wide antisense transcription in the pig genome and give an extensive new knowledge of the pig antisense transcription.

## Materials and Methods

### Ethics Statement

All animal work was conducted according to the guidelines for the care and use of experimental animals established by the Ministry of Agriculture of China. Animal Care and Use Committee (IACUC) in Jiangxi Agricultural University specifically approved this study.

### Animals and RNA Extraction

The White Duroc×Erhualian F_2_ resource population was created and managed as described by Guo et al. (2009) [47]. Briefly, 2 White Duroc boars and 17 Erhualian sows were crossed as founder animals to produce F_1_ animals, and 59 F_1_ sows were randomly mated with 9 F_1_ boars to generate 1,912 F_2_ individuals. At the age of 240±3 days, 1,030 F_2_ animals were slaughtered following Chinese industry standards.

A total of 497 liver and 586 *longissimus dorsi* muscle samples were harvested from the F_2_ population for RNA extraction. Total RNA was extracted using Trizol (Qiagen) according to the manufacturer’s protocol and then treated with DNase (New England Biolabs) for 30 min at 37°C to remove potential genomic DNA contamination. The quality of total RNA was assessed by an Agilent 2100 Bioanalyzer and 1% agarose gel electrophoresis.

### Library Construction and Sequencing

All libraries were constructed for DGE analyses following the method described in Morrissy et al. (2009) [30]. In brief, total RNA was used to isolate mRNA with the magnetic oligo (dT) beads (invitrogen). Double-stranded cDNA was synthesized using oligo-d (T) primers with the mRNA attached to the bead as a template. After digested with restriction enzyme *NlaIII* and *MmeI* (New England Biolabs), cDNA was ligated to Illumina specific adapters 1 and 2. Polymerase chain reaction (PCR) was used to enrich the cDNA library with two primers that annealed to the ends of the adapters. After purification and denaturation, the single chain molecules of each cDNA library were loaded onto the flowcell and sequenced on Illumina GA II.

### Tag Mapping, Annotation and Normalization

The data set of tags was analyzed according to the BGI bioinformatic protocols for DGE. In brief, the raw tags were first filtered to produce the clean tag data. For mapping clean tags to reference transcript sets or to the pig reference genome, we created virtual libraries containing all the possible 17-base length sequences of these resources located next to an *NlaIII* restriction site. The reference transcript sets were downloaded from the database of PEDE and pig unigene in NCBI. The redundant transcripts overlapped in two databases were removed from reference transcript set. For monitoring the mapping events on both strands, virtual sense and antisense tag sequence databases were generated for both full gene and cDNA sequences using in-house Perl scripts. The clean tag sequences were then mapped using SOAP2 [48] allowing up to one mismatches in 21-bp tag sequences. Sense and antisense tag sequences that unsuccessfully mapped to reference transcripts or mapped to multiple genes were filtered.

The number of clean tags that uniquely mapped to the reference transcript sequence of each gene was calculated and then normalized to TPM (number of tags mapped to each gene per million clean tags) as expression level of transcript.

### Genome-wide SNP Genotyping

The F_2_ animals were genotyped with Porcine SNP60 BeadChip following the *Infinium HD Assay Ultra* protocol (Illumina). The quality control of genotypes was carried out with GenABEL procedure in R. SNPs with call rates <95%, minor allele frequencies <1%, Hardy Weinberg equilibrium (HWE) *P* - value <5×10^−6^, or the X-linked SNPs that were likely to be autosomal (odds >1000 ) were excluded from further analysis.

### Strand-specific Real-time Quantitative RT-PCR

Total RNA was extracted from each 5 randomly selected liver and muscle samples, and then purified as described above. To confirm the DGE results, seven genes were randomly selected to verify their expression levels of sense and antisense transcripts in *longissimus dorsi* muscle and liver by strand-specific qRT-PCR according to the protocol described in Haddad et al. (2007) [49]. In brief, reverse transcription was performed on each RNA sample with strand-specific primers (Table S5) reverse complementary to target RNA and the Omniscript RT Kit (Qiagen). Negative controls to check for genomic DNA contamination (or for reagent purity) and for reverse transcriptase specificity were performed using the methods described in Haddad et al. (2007) [49]. Primers for real-time PCR were designed with Primer Express 3.0 (Applied Biosystems) (Table S5). Real-time PCR was performed in a 10 µl reaction mix including 0.5 µl cDNA, 0.2 µmol forward and reverse primer, and 5 µl Power SYBR Green PCR Master Mix using a 7900HT Real-Time PCR System (Applied Biosystems). The quantification of sense and antisense transcripts was determined by the comparative Ct method (2-ΔΔCt). The β-actin gene was chosen as an internal control and its real-time PCR was carried out using the cDNA synthesized with oligo dT primers. All samples were analyzed in three triplicates.

### Statistics

The hypergeometric test was applied to map all genes with antisense expression to terms in GO database and search significantly enriched GO terms comparing to the genomic background using DAVID Bioinformatics Resources 6.7 [50]. Multiple tests were corrected by FDR correction and enrichment threshold was set as EASE score of adjusted FDR *P*≤0.05. To analyze the correlation between sense and antisense expression of sense-antisense pairs and gene expression variability of sense and antisense transcripts, we corrected the effects of sex, batch and kinship on gene expression using R software. The means and standard deviations of expression levels of sense and antisense transcripts were calculated with R. To map the antisense eQTL, the expression values of antisense transcripts were corrected for sex, batch and kinship using R. In addition, considering the interaction between sense and antisense expression, the expression levels of sense transcripts were chosen as a covariant in antisense eQTL mapping. The associations between SNP genotypes and the expression levels of antisense transcripts were assessed using a linear regression model: Y = Xg, where Y is a vector of corrected expression value of an antisense transcript, and X is a vector of genotypes of a SNP, and g is the additive effect of the SNP. Genome-wide significant thresholds were adjusted by FDR and the significant threshold was set at FDR <0.05. All calculations were executed by the R software package (V2.13.0).

## Supporting Information

Table S1 GO functional annotation of genes with antisense expression.
(XLS)

Table S2 The normalized expression level of sense and antisense transcript of the imprinting genes in liver and muscle.
(XLS)

Table S3 The eQTL mapping results of antisense transcripts in liver and muscle.
(XLS)

Table S4 Transcripts having both sense and antisense eQTL.
(XLS)

Table S5 Primers for strand-specific reverse transcription and real-time PCR.
(XLS)
